# Sociodemographic and Clinical Factors for Microcephaly Secondary to Teratogenic Infections in Brazil: An Ecological Study

**DOI:** 10.3390/v15081675

**Published:** 2023-07-31

**Authors:** Arlison Pereira Ferreira, Davi Silva Santana, Eric Renato Lima Figueiredo, Marcelo Coelho Simões, Dionei Freitas de Morais, Victória Brioso Tavares, Juliana Gonçalves de Sousa, Marcos Jessé Abrahão Silva, Fabiana de Campos Gomes, João Simão de Melo Neto

**Affiliations:** 1 Unidade de Pesquisa Clínica e Experimental do Sistema Urogenital (UPCEURG), Instituto de Ciências da Saúde, Universidade Federal do Pará (UFPA), Belém 66075-110, PA, Brazil; arlisonferreira.bio@gmail.com (A.P.F.); davi.santana@ics.ufpa.br (D.S.S.); eric.renatoo@gmail.com (E.R.L.F.); victoria.tavares15@gmail.com (V.B.T.); julianasousa0419@gmail.com (J.G.d.S.); 2 Programa de Pós-Graduação em Ciências Ambientais, Universidade do Estado do Pará (UEPA), Belém 66095-100, PA, Brazil; marcelo.uepa14@gmail.com; 3 Faculdade de Medicina de São José do Rio Preto (FAMERP), São José do Rio Preto 15090-000, SP, Brazil; dionei.fm@terra.com.br; 4 Instituto Evandro Chagas (IEC), Ananindeua 67030-000, PA, Brazil; jesseabrahao10@gmail.com; 5 Faculdade de Medicina Ceres (FACERES), São José do Rio Preto 15090-305, SP, Brazil; facamposgomes@gmail.com

**Keywords:** epidemiology, infant, newborn, microcephaly, syphilis, toxoplasmosis, Zika virus

## Abstract

Microcephaly is a neurological condition characterized by anomalies in the growth of the cranial circumference. This study aims to examine the association between sociodemographic and clinical variables and the occurrence of secondary microcephaly in newborns in Brazil. It also aims to investigate the association between this congenital anomaly and teratogenic infections. This research adopts an observational approach with an ecological, descriptive, and analytical design. The sample includes infants aged ≤28 days and registered in the country’s Live Births Information System from January 2015 to December 2021. Newborns were categorized into G1, consisting of newborns with one of the three infections (Zika, toxoplasmosis, or syphilis), and G2, consisting of newborns with two of the three infections. A total of 1513 samples were analyzed and divided into two groups: one infection (syphilis n = 423; toxoplasmosis n = 295; or Zika n = 739) and two infections (n = 56). The northeastern region of Brazil has the highest prevalence of microcephaly. Regarding the population profile, the Zika virus infection is more common among white mothers, while the syphilis infection is more common among black mothers. Among newborns with microcephaly, boys have a lower prevalence of toxoplasmosis infection, while girls have a lower prevalence of Zika virus infection. This study provides pertinent information on each infection and contributes to the epidemiologic understanding of the association between teratogenic infections and microcephaly.

## 1. Introduction

Microcephaly is a neurological condition characterized by anomalies in the growth of the cranial circumference, which can be identified by abnormalities in the reduction of measurements used to estimate this growth [1]. These congenital abnormalities can result from neurogenesis, synaptogenesis, and neuronal migration, leading to macroscopic central nervous system (CNS) effects and brain parenchymal calcifications [2]. According to the World Health Organization (WHO), the cranial circumference (CP) in these cases is less than minus two (−2) standard deviations from the sex- and gestational-age-specific mean, and the presence of a value less than minus three (−3) standard deviations indicates severe microcephaly [3,4]. The main causes of microcephaly are genetic, perinatal, postnatal, and environmental factors. Depending on its origin, microcephaly can be classified as primary, in which malformations occur in the early months of pregnancy due to genetic/chromosomal and/or environmental abnormalities, or secondary, initially characterized by normal CP and later by disruption of normal head growth [5] resulting from interactions with environmental factors, such as congenital infections that have secondary effects on the developing inflammatory process and teratogenic reactions affecting CP [6].

The major congenital infections associated with the clinical presentation studied in this study are collectively referred to as TORCH, an acronym for toxoplasmosis, rubella, cytomegalovirus, herpes simplex infection, syphilis, and acquired immunodeficiency syndrome (AIDS) [6,7,8]. Transplacental transmission is the primary mode of transmission for these diseases. Congenital infections manifest clinically in the fetus in a similar manner, causing nonprogressive neurologic disorders, and congenital and perinatal infections that are the leading cause of childhood disability worldwide [9]. In addition, congenital toxoplasmosis, in its classical form, can lead to the tetrad of clinical manifestations: chorioretinitis, hydrocephalus/microcephaly, cerebral calcifications, and neurological changes [10]. According to a recent study in a large cohort, the incidences of seroconversion and presumed toxoplasmosis infection during pregnancy were 0.8 per 1000 live births, whereas the incidences of congenital toxoplasmosis were 0.1 per 1000 live births. In addition, the presence of maternal symptoms, such as lymphadenopathy, was associated with a higher risk of congenital infection [11,12]. Studies in Brazil showed a Zika rate of 6.7 per 10,000 live births [13] and a moving average of 3.92 per 1000 live births for syphilis [14].

Strengthening the health system to control and prevent congenital infections through public health strategies can help prevent the occurrence of microcephaly associated with these infections. However, this problem persists in some developing and underdeveloped countries, resulting in high rates of morbidity in the population [15].

In Brazil, after identifying an increase in microcephaly cases in Pernambuco, the government launched an epidemiological investigation in October 2015 to analyze whether the increase was a fluctuation in numbers or a potential outbreak. A preliminary analysis of the Live Birth Information System (SINASC) showed that the numbers observed in Pernambuco in October 2015 were significantly higher than usual. In response, the WHO and the Brazilian government declared the microcephaly epidemic a national and international public health emergency in November 2015 [16,17,18].

ZIKV is transmitted by Aedes mosquitoes and can cause severe central nervous system complications, including microcephaly. Microcephaly is a condition in which a baby’s head is smaller than normal. It can cause a range of health problems, including developmental delays, seizures, and intellectual disabilities [19,20].

In addition to ZIKV, other infections can cause microcephaly, including syphilis and toxoplasmosis. Syphilis is a sexually transmitted infection that can be passed from mother to fetus during pregnancy. Toxoplasmosis is an infection caused by a parasite that can be transmitted by eating raw or undercooked meat or by contact with the urine of infected cats [21,22,23,24].

This scenario should also be studied in the context of the sociodemographic characteristics of each locality and their association with microcephaly, especially in developing countries. Therefore, the aim of this study is to investigate the association between sociodemographic and clinical variables and the occurrence of secondary microcephaly in newborns in Brazil. It is also intended to investigate the association between this congenital anomaly and teratogenic infections. The study hypothesis is whether there are significant differences in sociodemographic and clinical variables related to microcephaly caused by teratogenic infections alone or in co-infection in Brazil.

## 2. Material and Methods

### 2.1. Ethical Considerations

The data used are limited to secondary variables and do not reveal the personal data of the patients, therefore, in compliance with the National Health Council (CNS) Resolution No. 510 of 7 April 2016, the study was exempted from the mandatory evaluation by the corresponding research ethics committee [25].

### 2.2. Type of Study

This observational study with an ecological design includes descriptive and inferential analyzes of live births of both sexes diagnosed with microcephaly between 2015 and 2021 in Brazil.

### 2.3. Data Base

The study used data from the SINASC [26], in this health information system, the registration of live births occurring in the country is carried out to support public policies and gain knowledge of the population’s health status related to births, these data include mandatory registration information for users of the country’s universal health system [27,28].

### 2.4. Description and Characterization of the Area

The study was conducted in Brazil, divided into five macroregions: north, northeast, midwest, southeast, and south. According to the Brazilian Institute of Geography and Statistics (IBGE), the country has a total area of 8,510,345.540 km^2^, composed of 5570 municipalities, and an estimated population of 213,317,639 inhabitants in 2021, corresponding to a population density of 22.43 inhabitants per km^2^, an average fertility rate of 1.76 children per woman, and an infant mortality rate of 11.56 deaths per thousand live births, one of the highest rates in Latin America [29].

### 2.5. Population and Study Period 

Live newborns were identified according to the International Statistical Classification of Diseases and Related Health Problems (ICD-10), which are selected newborns with ICD-10 code Q02, corresponding to microcephaly within the neonatal period defined as 28 days of age or less. The time frame for this study was set from 2015 to 2021. This time frame is justified by the World Health Organization’s confirmation of congenital Zika syndrome (CZS) in the northeastern region of Brazil, the identification of its association with Guillain-Barré syndrome and central nervous system malformations at birth, and the start of international reporting in other countries in the Americas in 2015 [30]. The selected time period corresponds to the last complete annual report available in the SINASC database. Data that were missing from the database, indicating gaps in the completion of the SINASC system, were excluded. Specifically, there was only one case with the three congenital infections, configuring a non-representative and excluded sample. In addition, the year 2022 was excluded from the analysis, as it does not provide a complete overview of the consolidated data in the said platform. Newborns with congenital anomalies other than microcephaly were filtered out and subsequently excluded from the analysis in order to focus the panorama only on secondary microcephaly in the country.

### 2.6. Allocation of the Selected Samples

**Figure 1:**
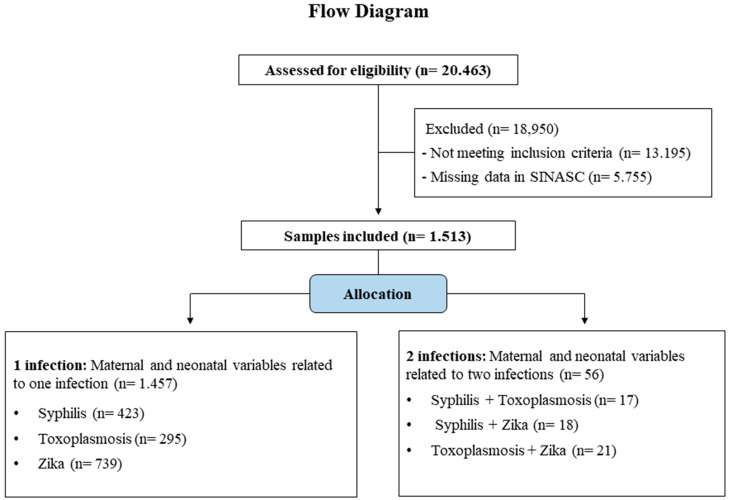
Flow diagram of sample eligibility and group allocation.

Figure 1 illustrates the sample selection procedure, which followed the CONSORT templates of the Equatorial Network International Observational Project [31]. The newborns were divided into two groups: Group 1 infection (neonates ≤ 28 days of age with one secondary microcephaly related infection) and Group 2 infection (neonates ≤ 28 days of age with two secondary microcephaly related infections).

### 2.7. Variables

The variables were divided into two categories: sociodemographic and clinical factors (maternal and neonatal). Accordingly, the sociodemographic context in this study was examined through the description of maternal race/ethnicity, neonatal sex, birth year, and live birth classification. Maternal clinical factors were assessed based on maternal age, type of pregnancy, gestational fever, exanthema, pruritus, conjunctivitis, joint pain, muscle pain, edema, headache, ganglionic hypertrophy, and nerve damage. On the other hand, neonatal clinical factors were determined by weight, head circumference, neurological impairment, visual impairment, hearing impairment, type of evidence, and imaging findings (magnetic resonance imaging, trans fontanel ultrasonography, computed tomography, and ultrasonography).

### 2.8. Statistical Analysis

Data were organized in Microsoft Excel 2016 and then transferred to the Statistical Package for Social Science for Windows (IBM SPSS) software (version 21). The normality of the data was assessed using the Kolmogorov–Smirnov test, considering that all samples had representative values greater than 50. Descriptive analysis was performed to obtain frequency values (absolute and relative), mean and standard deviation (for parametric variables), and median with interquartile range (IQR, for nonparametric variables) for the groups. For parametric variables with more than two groups, a one-way analysis of variance (ANOVA) was used, followed by Tukey’s post-test for significance analysis. For nonparametric independent sample data with more than two groups, the Kruskal–Wallis one-factor test was used, along with a pairwise comparison (for scalar variables) and a Pearson’s chi-squared or Fisher’s exact test for categorical variables. Values were adjusted using Bonferroni’s method for comparing proportions to identify significant differences between variables within each group. For segments with two or fewer groups, the Mann–Whitney test (nonparametric) and the *t*-test (parametric) for independent samples were used. Statistical significance was defined as a *p*-value of <0.05.

## 3. Results

### 3.1. Neonatal and Maternal Sociodemographic Factors

A total of 1457 cases were analyzed, divided into three different infection groups: syphilis (423), toxoplasmosis (295), and Zika virus (739). In the sociodemographic analysis (Table 1), self-reported white mothers predominated with Zika virus infections (30.3%) compared to syphilis infections (19.8%). Similarly, self-reported black mothers predominated with syphilis infections (15.1%) compared to Zika virus infections (4.8%) (*p* < 0.050). Among male newborns, the prevalence of toxoplasmosis (37.9%) was lower than that of the Zika virus (47.9%), but among female newborns, the prevalence of toxoplasmosis (62.1%) was higher than that of the Zika virus (52.1%) (*p* = 0.006). 

Considering the year of birth of the newborns, the years 2015 and 2016 showed a higher prevalence of Zika virus infections (29.0%, 37.8%) compared to the other years (*p* < 0.001). From 2018 to 2021, the prevalence of Zika virus infections became lower compared to the other years (8.5%, 5.4%, 4.2%, and 2.6%). When classifying live births by gestational age, preterm deliveries were more common with toxoplasmosis infections (29.1%) compared to Zika virus infections (20.0%) (*p* = 0.025).

**Figure 2:**
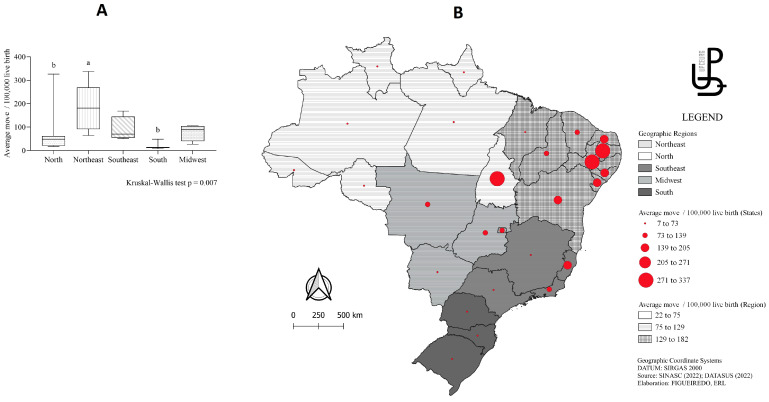
Moving average of microcephaly incidence in different regions of Brazil (**A**) and in federal entities (**B**). ^a,b^ *p* < 0.05, Dunn’s post hoc test.

Table 2 shows the comparison between groups with one or two infections. Only the year of birth variable showed a difference between groups (*p* = 0.019), with a higher prevalence of live births with one infection (20.9%) compared to two infections (9.1%) in 2015 (*p* = 0.019). The average incidence rate of newborns younger than 28 days diagnosed with microcephaly per 100,000 live births by Brazilian region and state between 2015 and 2021 is shown in Figure 2A. In descending order, the highest moving average of microcephaly incidence per year was diagnosed in the northeast region (182), followed by the southeast (90), north (80), midwest (78) and south (23). Among the states, the highest moving averages were found in Pernambuco (337), Paraíba (335), Tocantins (326), Alagoas (203), Rio Grande do Norte (196), Bahia (181), and Espírito Santo (168) (Figure 2B).

### 3.2. Maternal Clinical Factors

Table 3 presents data from the analysis of maternal clinical factors associated with the development of secondary microcephaly. Maternal age was lower for syphilis (mean = 25, SD = 11) and toxoplasmosis (mean = 26, SD = 11) infections than for Zika virus infections (28) (*p* < 0.001). Pregnancy fever (*p* < 0.001) was most common with Zika virus infections (48.8%) compared to syphilis (14.7%) and toxoplasmosis (14.9%). Exanthema during pregnancy (*p* < 0.001) was more common with Zika virus infections in the first (41.4%), second (18.2%), and third (8.8%) trimesters compared to other infections. Other pregnancy manifestations also showed statistical significance. Among them, pruritus (*p* < 0.001), conjunctivitis (*p* < 0.001), arthralgia (*p* < 0.001), myalgia (*p* < 0.001), edema (*p* = 0.034), headache (*p* < 0.001), and ganglionic hypertrophy (*p* = 0.032) were more common with Zika virus infections compared to the others. From another perspective, Table 4 shows the results of the analysis comparing the profiles of one or two infections. Maternal age (*p* = 0.012) was higher in mothers with one infection (mean = 27, SD = 10) than in mothers with two infections (mean = 23, SD = 9).

### 3.3. Clinical Factors in Newborns

Table 5 shows the results of the clinical analysis of the neonates for each infection group. The newborn weight was higher (mean = 2660.00, SD = 728, *p* = 0.001), and the head circumference was lower (mean = 30.00, SD = 4, *p* = 0.491) in those with the Zika virus infection. Diagnosis during pregnancy was more common with the Zika virus infection (43.5%) and postpartum in the syphilis infection (82.7%) (*p* < 0.001). MRI findings suggestive of congenital infection were more common with the Zika virus infection (84.2%) compared to toxoplasmosis (0%), while findings suggestive of other changes were more common with the toxoplasmosis infection (100%) compared to the Zika virus infection (0%) (*p* = 0.002). With regard to trans-fontanel ultrasonography (*p* < 0.001), normal results were more common with toxoplasmosis (83.3%) and Zika virus (76.9%) infections than with syphilis (41.9%) infections. Among altered results suggestive of congenital infections, the prevalence was lower for the toxoplasmosis (11.7%) and Zika virus (15.6%) infections than for syphilis (46.5%) infections. In computed tomography scan analyzes (*p* < 0.001), normal results (23.1%) were less frequent, while altered results suggestive of congenital infection (71.4%) were more frequent in the syphilis infections compared to the others. Among altered results with other abnormalities, a higher prevalence was observed for the Zika virus (11.8%) compared to syphilis (4.5%).

Abnormal results suggested infection was more common for syphilis (37.6%) than for toxoplasmosis (12.8%) (*p* = 0.007). Table 6, which shows the results of neonatal clinical factors comparing one or two infections, did not show statistically significant data.

## 4. Discussion

According to the results presented, this study accepts the alternative hypothesis, since there are significant differences in sociodemographic and clinical variables related to microcephaly caused by teratogenic infections alone or in co-infection in Brazil.

Regarding the geo-referenced socio-demographic factors in the study, it was observed that the highest frequency of these diseases occurred in the northeastern region during the period analyzed. This may be partly due to the fact that it is a tourist region, which contributes to a significant influx of people from different parts of the country and the world, making these populations more vulnerable to transmission; as a result, public health authorities have initiated epidemiological investigations to identify the emergence and possible links [31].

The incidence of microcephaly was higher among non-white mothers compared to black mothers. This finding regarding race/color may be attributed to factors such as low education, inadequate income, living in disadvantaged areas, and poor neighborhoods [32]. These factors may create barriers to accessing health care services and obtaining means of STI prevention. Consequently, they affect the higher prevalence of syphilis infections among self-reported black mothers. Syphilis infection has a longer incubation period compared to Zika virus, leading to potential negative consequences if the disease is diagnosed late in pregnant women [33].

We did not uncover pertinent findings that elucidate the exact association between maternal age and birth year; however, it is plausible that these variables are important in the epidemiology of microcephaly, especially in the context of Brazil. This speculation stems from the declaration of a public health emergency in November 2015, which triggered a notable increase in disease surveillance and prevention efforts between 2010 and 2017 [34].

Male newborns had a higher prevalence of toxoplasmosis, while female newborns had a lower prevalence. It has been observed that the toxoplasma gondii infection can affect male viability and survival in humans, which may be related to hormone levels [35]. Therefore, females may be more susceptible to developing microcephaly, as observed in this study. In the case of the Zika virus, the gender distribution was balanced.

In 2015 and 2016, Brazil experienced an outbreak of microcephaly cases associated with ZIKV [36], as confirmed by the results of this study, which showed a higher number of cases with a single infection. Several hypotheses have been proposed to explain this outbreak, but there is still controversy among experts in the field. However, all hypotheses relate to the introduction of the virus to Brazil through the large movement of people during sporting events [37]. Health authorities responded intensively, resulting in a subsequent decrease in Zika cases [38]. In 2018 and 2019, syphilis infections were more frequent. However, with the onset of the COVID-19 pandemic, social isolation, and the use of personal protective equipment, an increase in the incidence of microcephaly secondary to toxoplasmosis infection was observed in 2020.

Toxoplasmosis infection has been associated with higher rates of preterm birth. Acute gestational toxoplasmosis can lead to a miscarriage or severe damage to the eyes, brain, and other structures of the fetus. Although the impact of toxoplasmosis on prematurity is not fully understood, it has been suggested that it may be related to the cascade of inflammatory events in the placenta and fetus [39]. Children with microcephaly showed weight loss, although the post hoc test did not show significance. Of the infections, the Zika virus infection had the least effect on weight loss. Microcephaly itself may directly contribute to lower birth weight by reducing cranial volume and brain weight [40]. However, accurate measurement of these parameters can be challenging.

Women in later stages of pregnancy presented with isolated Zika virus infection. A previous study [33] also found a higher prevalence in this population. However, there is no clear cause-and-effect relationship. Symptoms such as gestational fever, exanthema, pruritus, conjunctivitis, arthralgia, myalgia, edema, headache, and ganglion hypertrophy were more common in mothers with Zika virus. These symptoms have been previously associated with pathology [41], which facilitates the diagnostic process. Computed tomography, plain ultrasound, and trans-fontanel scans were more effective in diagnosing microcephaly in pregnant women with syphilis. However, although imaging tests may be influenced by other congenital infections, the absence of abnormalities does not rule out the presence of pathology. Infections can cause gradual or subtle deformities in neonatal anatomy that may not be apparent on such tests [42].

As a strength of this research, it highlights the national perspective on sociodemographic and clinical factors associated with one or more teratogenic infections associated with microcephaly in Brazil, while previous studies have focused on specific regions or states. As inherent limitations of an observational approach that incorporates an ecological design, we first emphasize that this specific design prevents the establishment of causal relationships between exposures and outcomes and instead provides associations and trends within the confines of the population level [43]. In addition, the use of data from the SINASC introduces potential biases, including the possibility of under-reporting or misclassification of infections [44]. The dichotomous classification of newborns into two distinct groups based on the presence of specific infections may oversimplify the complex nature of the interactions between these infections and their potential combined effects. In addition, the temporal scope of the study, from January 2015 to December 2021, may not capture recent changes in the epidemiologic landscape of these infections. Finally, the issue of generalizability should be considered when interpreting the results, as the study focused only on infants aged ≤28 days, which may limit its representativeness to the general population.

The results of this study showed that the Zika virus infection was more common among white mothers, while the syphilis infection was more common among black mothers. These ethnic/racial differences should be interpreted with the understanding that in Brazil, the classification of race/ethnicity by the Brazilian Institute of Geography and Statistics is based on self-report. This means that ethnic categories may not be accurate due to the lack of clear definitions of ethnic, linguistic, cultural, or historical characteristics [45]. 

The scientific literature also shows heterogeneity of associated sociodemographic factors [46], but socioeconomic status is an important determinant influencing these rates. [47] Poverty-related variables, such as low maternal education, maternal smoking during pregnancy, and preterm and vaginal birth, were generally more strongly associated with microcephaly in Brazil, Canada, and China [45,48,49].

## 5. Conclusions

The present study investigated the sociodemographic and clinical factors associated with microcephaly in live births in northeastern Brazil. The results showed that the northeastern region of Brazil had the highest incidence of microcephaly. Self-reported white mothers were more likely to have the Zika virus infection, while black mothers were more likely to have the syphilis infection. Toxoplasmosis had a lower prevalence in male newborns, while Zika virus had a lower prevalence in female newborns. In 2015 and 2016, the Zika virus was the main teratogenic factor, while syphilis predominated in 2018 and 2019, and toxoplasmosis emerged as the main factor in 2020. The toxoplasmosis infection was associated with higher rates of preterm birth. In 2015, there were more cases of a single infection. Women in later stages of pregnancy had isolated Zika virus infection. Symptoms such as gestational fever, exanthema, pruritus, conjunctivitis, arthralgia, myalgia, edema, headache, and ganglion hypertrophy were more common in mothers with the Zika virus. Children with microcephaly had weight loss, with the Zika virus having the least effect. Most cases of all infections were detected postpartum. CT scan, plain ultrasound, and trans fontanel examination were more accurate in diagnosing microcephaly in syphilis cases. The results of this study suggest that sociodemographic and clinical factors show variation among live births with microcephaly resulting from teratogenic infections caused by different microorganisms. The results also highlight the importance of early diagnosis and intervention for children with microcephaly.

## Figures and Tables

**Table 1:** Neonatal and maternal sociodemographic factors of secondary microcephaly related to different microorganisms.

	Syphilis (n = 423)	Toxoplasmosis (n = 295)	Zika Vírus (n = 739)	*χ*^2^ or Fisher’s Exact Test	*p*-Value
Race/Ethnicity Maternal					
White	64 (19.8%) ^a^	74 (31.4%)	127 (30.3%) ^b^	1.165 º	<0.050 *
Black	49 (15.1%) ^a^	20 (8.5%)	20 (4.8%) ^b^		
Yellow	1 (0.3%)	2 (0.8%)	3 (0.7%)		
Brown	208 (64.2%)	138 (58.5%)	268 (64%)		
Indigenous	2 (0.6%)	2 (0.8%)	1 (0.2%)		
Sex of the neonate					
Male	173 (41.2%)	111 (37.9%) ^a^	348 (47.9%) ^b^	10.206 ^#^	0.006 *
Female	247 (41.2%)	182 (62.1%) ^a^	379 (52.1%) ^b^		
Year of birth					
2015	53 (12.6%) ^a^	36 (12.3%) ^a^	214 (29%) ^b^	131.829 ^#^	<0.001 *
2016	127 (30.2%)	75 (25.6%) ^a^	279 (37.8%) ^b^		
2017	59 (14%)	48 (16.4%)	92 (12.5%)		
2018	59 (14%) ^a^	38 (13%)	63 (8.5%) ^b^		
2019	50 (11.9%) ^a^	40 (13.7%) ^a^	40 (5.4%) ^b^		
2020	37 (8.8%)	36 (12.3%) ^a^	31 (4.2%) ^b^		
2021	35 (8.3%) ^a^	20 (6.8%)	19 (2.6%) ^b^		
Classification of the live birth					
Preterm birth (<37 weeks)	100 (24.5%)	82 (29.1%) ^a^	134 (20%) ^b^	5.017 º	0.025 *
Term birth (37 to <42 weeks)	307 (75.2%)	199 (70.6%) ^a^	529 (79%) ^b^		
Post term birth (≥42 weeks)	1 (0.2%)	1 (0.4%)	7 (1%)		

(^a,b^) Values with significant differences in the post test; (*) Significant values are represented with *p* < 0.050; (^#^) Pearson’s chi-square; (º) Fisher’s Exact Test; The missing values in the total sum of categories and groups correspond to the values omitted from the databases.

**Table 2:** Maternal and neonatal sociodemographic factors in microcephaly secondary to teratogenic infections arising from one or more infections.

	1 Infection (n= 1457 )	2 Infections (n = 56 )	*χ*^2^ or Fisher’s Exact Test	*p*-Value
Race/Ethnicity			0.923 º	0.337
White	265 (27.1%)	9 (20.5%)		
Black	89 (9.1%)	4 (9.1%)		
Yellow	6 (0.6%)	0 (0%)		
Brown	614 (62.7%)	31 (70.5%)		
Indigenous	5 (0.5%)	0 (0%)		
Sex of the neonate			0.409 ^#^	0.261
Male	632 (43.9%)	27 (48.2%)		
Female	808 (56.1%)	29 (51.8%)		
Year of birth			5.472 º	0.019 *
2015	303 (20.9%) ^a^	5 (9.1%) ^b^		
2016	481 (33.1%)	17 (30.9%)		
2017	199 (13.7%)	10 (18.2%)		
2018	160 (11%)	9 (16.4%)		
2019	130 (9%)	1 (1.8%)		
2020	104 (7.2%)	7 (12.7%)		
2021	74 (5.1%)	6 (10.9%)		
Classification of the live birth			0.387 ^#^	0.412
Preterm birth (<37 weeks)	316 (23.2%)	12 (25%)		
Term birth (37 to <42 weeks)	1035 (76.1%)	36 (75%)		
Post term birth (≥42 weeks)	9 (0.7%)	0 (0%)		

(^a,b^) Values with significant differences in the post test; (*) Significant values are represented with *p* < 0.050; (^#^) Pearson’s chi-square; (º) Fisher’s Exact Test; The missing values in the total sum of categories and groups correspond to the values omitted from the databases.

**Table 3:** Maternal clinical factors of secondary microcephaly related to different microorganisms.

	Syphilis (n = 419)	Toxoplasmosis (n = 275)	Zika Vírus (n = 727)	*χ*^2^, Fisher’s Exact Test or H Value	*p*-Value
Mother’s Age §	25 (11) ^a^	26 (11) ^a^	28 (10) ^b^	2316 ¢	<0.001 *
Type of pregnancy					
Single	393 (96.1%)	279 (98.2%)	655 (97.5%)		
Double	16 (3.9%)	5 (1.8%)	16 (2.4%)	1009 º	0.315
Triple	0 (0%)	0 (0%)	0 (0%)		
Gestational fever					
Yes	48 (14.7%) ^a^	35 (14.9%) ^a^	264 (48.8%) ^b^	148.04 ^#^	<0.001 *
No	279 (85.3%) ^a^	200 (85.1%) ^a^	277 (51.2%) ^b^		
Exanthema					
Yes, in the 1st quarter	23 (7.2%) ^a^	23 (10%) ^a^	239 (41.4%) ^b^		
Yes, no 2nd quarter	12 (3.8%) ^a^	9 (3.9%) ^a^	105 (18.2%)^b^		
Yes, no 3rd trimester	7 (2.2%) ^a^	5 (2.2%) ^a^	51 (8.8%) ^b^	354.9 ^#^	<0.001 *
Yes, but no gestational date or period	10 (3.1%)	4 (1.7%)	22 (3.8%)		
Exanthema absent	267 (83.7%) ^a^	188 (82.1%) ^a^	160 (27.7%) ^b^		
Pruritus					
Yes	25 (5.9%) ^a^	17 (5.8%) ^a^	177 (24%) ^b^	93.436 ^#^	<0.001 *
No	398 (94.1%) ^a^	278 (94.2%) ^a^	562 (76%) ^b^		
Conjunctivitis					
Yes	8 (1.9%) ^a^	1 (0.3%) ^a^	41 (5.5%) ^b^	21.531 ^#^	<0.001 *
No	415 (98.1%) ^a^	294 (99.7%) ^a^	698 (94.5%) ^b^		
Joint pain					
Yes	20 (4.7%) ^a^	5 (1.7%) ^a^	135 (18.3%) ^b^	83.082 ^#^	<0.001 *
No	403 (95.3%) ^a^	290 (98.3%) ^a^	604 (81.7%) ^b^		
Muscular pain					
Yes	24 (5.7%) ^a^	5 (1.7%) ^b^	105 (14.2%) ^c^	48.39 ^#^	<0.001 *
No	399 (94.3%) ^a^	290 (98.7%) ^b^	634 (85.8%) ^c^		
Edema					
Yes	6 (1.4%) ^a^	2 (0.7%) ^a^	22 (3%) ^b^	6739 ^#^	0.034 *
No	417 (98.6%) ^a^	293 (99.3%) ^a^	717 (97%) ^b^		
Headache					
Yes	29 (6.9%) ^a^	22 (7.5%) ^a^	128 (17.3%) ^b^	35.34 ^#^	<0.001 *
No	394 (93.1%) ^a^	273 (92.5%) ^a^	611 (82.7%) ^b^		
Ganglion hypertrophy					
Yes	1 (0.2%) ^a^	1 (0.3%) ^a^	10 (1.4%) ^b^	4579 º	0.032 *
No	422 (99.8%) ^a^	294 (99.7%) ^a^	729 (98.6%) ^b^		
Neural damage					
Yes	3 (0.7%)	0 (0%)	4 (0.5%)	0.051 º	0.821
No	420 (99.3%)	295 (100%)	735 (99.5%)		

(^a,b^) Values with significant differences in the post test; (*) Significant values are represented with *p* < 0.050; (§) Kruskal–Wallis 1-way ANOVA pairwise; (¢) Kruskal–Wallis H; (^#^) Pearson’s chi-square; (º) Fisher’s Exact Test; The missing values in the total sum of categories and groups correspond to the values omitted from the databases.

**Table 4:** Maternal clinical factors in microcephaly secondary to teratogenic infections arising from one or more infections.

	1 Infection (n = 1457)	2 Infections (n = 67)	*χ*^2^, Fisher’s Exact Test or Mann–Whitney U	*p*-Value
Mother’s Age ¬	27 (10) ^a^	23 (9) ^b^	28.34000	0.012 *
Type of Pregnancy				
Single	1327 (97.2%)	47 (97.9%)	0.096 º	0.757
Double	37 (2.7%)	1 (2.1%)		
Triple	1 (0.1%)	0 (0%)		
Gestational fever				
Yes	374 (31.5%)	13 (26.0%)	0.664 ^#^	0.415
No	756 (68.5%)	37 (74%)		
Exanthema				
Yes, in the 1st quarter	285 (25.3%)	11 (22.9%)		
Yes, no 2nd quarter	126 (11.2%)	6 (12.5%)	0.614 ^#^	0.958
Yes, no 3rd trimester	63 (5.6%)	2 (4.2%)		
Yes, but no gestational date or period	36 (3.2%)	1 (2.1%)		
Exanthema absent	615 (54.7%)	28 (58.3%)		
Pruritus				
Yes	219 (15%)	6 (10.7%)	0.794 ^#^	0.373
No	1238 (85%)	50 (89.3%)		
Conjunctivitis				
Yes	50 (3.4%)	2 (3.6%)	0.003 º	0.955
No	1407 (96.6%)	54 (96.4%)		
Joint pain				
Yes	160 (11%)	7 (12.5%)	0.127 ^#^	0.722
No	1297 (89%)	49 (87.5%)		
Muscular pain				
Yes	134 (9.2%)	3 (5.4%)	0.966^#^	0.326
No	1323 (90.8%)	53 (94.6%)		
Edema				
Yes	30 (2.1%)	1 (1.8%)	0.020 º	0.887
No	1427 (97.9%)	55 (98.2%)		
Headache				
Yes	179 (12.3%)	7 (12.5%)	0.002 ^#^	0.962
No	1278 (87.8%)	49 (87.5%)		
Ganglion hypertrophy				
Yes	12 (0.8%)	0 (0%)	0.465 º	0.495
No	1445 (99.2%)	56 (100%)		
Neural damage				
Yes	7 (0.5%)	0 (0%)	0.270 º	0.603
No	1450 (99.5%)	56 (100%)		

(^a,b^) Values with significant differences in the post test; (*) Significant values are represented with *p* < 0.050; (¬) Mann–Whitney U-test; (^#^) Pearson’s chi-square; (º) Fisher’s Exact Test; The missing values in the total sum of categories and groups correspond to the values omitted from the databases.

**Table 5:** Clinical factors in newborns with secondary microcephaly related to different microorganisms.

	Syphilis (n = 419)	Toxoplasmosis (n = 275)	Zika Vírus (n = 727)	*χ*^2^, Fisher’s Exact Test or H Value	*p*-Value
Newborn weight §	2347.5 (636)	2435 (760)	2660 (728)	3197 ¢	<0.001 *
Cephalic Perimeter §	30.50 (2)	30.40 (3)	30.00 (4)	1611 ¢	0.491 *
Neurological Disability					
Yes	5 (1.2%)	4 (1.4%)	13 (1.8%)	0.661 ^#^	0.718
No	418 (98.8%)	291 (99.6%)	726 (98.2%)		
Visual Impairment					
Yes	2 (0.5%)	3 (1.0%)	5 (0.7%)	0.093 º	0.761
No	421 (99.5%)	292 (99.5%)	734 (99.3%)		
Hearing Impairment					
Yes	7 (1.7%)	7 (2.4%)	15 (2%)	0.471 ^#^	0.790
No	416 (98.3%)	288 (97.6%)	724 (98%)		
Detection Type					
Intrauterine (during pregnancy)	68 (17.3%)	68 (25.6%)	191 (43.5%)	71.08 ^#^	<0.001 *
Postpartum	325 (82.7%)	198 (74.4%)	248 (56.5%)		
Magnetic resonance imaging results					
Normal result	2 (11.1%)	0 (0%)	2 (10.5%)		
Altered result, suggestive of infection	12 (66.7%)	0 (0%) ^a^	16 (84.2%) ^b^	20.5 ^#^	0.002 *
Altered result, with other changes	3 (16.7%)	3 (100%) ^a^	0 (0%) ^b^		
Indeterminate result	1 (5.6%)	0 (0%)	1 (5.3%)		
Ultrasonography Transfontanela Result					
Normal result	36 (41.9%) ^a^	50 (83.3%) ^b^	123 (76.9%) ^b^		
Altered result, suggestive of congenital infection	40 (46.5%) ^a^	7 (11.7%) ^b^	25 (15.6%) ^b^	41.95 ^#^	<0.001 *
Altered result, with other changes	8 (9.3%)	2 (3.3%)	9 (5.6%)		
Indeterminate result	2 (2.3%)	1 (1.7%)	3 (1.9%)		
CT scan result					
Normal result	46 (23.1%) ^a^	55 (62.5%) ^b^	136 (53.3%) ^b^		
Altered result, suggestive of congenital infection	142 (71.4%) ^a^	26 (29.5%) ^b^	84 (32.9%) ^b^	82.86 ^#^	<0.001 *
Altered result, with other changes	9 (4.5%) ^a^	7 (8%)	30 (11.8%) ^b^		
Indeterminate result	2 (1%)	0 (0%)	5 (2%)		
Ultrasonography result					
Normal result	34 (40%) ^a^	34 (72.3%) ^b^	48 (56.5%)		
Altered result, suggestive of infection	32 (37.6%) ^a^	6 (12.8%) ^b^	16 (18.8%)	17.774 ^#^	0.007 *
Altered result, with other changes	9 (10.6%)	2 (4.3%)	10 (11.8%)		
Indeterminate result	10 (11.8%)	5 (10.6%)	11 (12.9%)		

(^a,b^) Values with significant differences in the post test; (*) Significant values are represented with *p* < 0.050; (§) Kruskal–Wallis 1-way ANOVA pairwise; (¢) Kruskal–Wallis H; (^#^) Pearson’s chi-square; (º) Fisher’s Exact Test; The missing values in the total sum of categories and groups correspond to the values omitted from the databases.

**Table 6:** Neonatal clinical factors in microcephaly secondary to teratogenic infections arising from one or more infections.

	1 Infection (n = 1457)	2 Infections (n = 56)	*χ*^2^, Fisher’s Exact Test or Mann–Whitney U	*p*-Value
Newborn weight ¬	2520 (726)	2655 (870)	286.443.500	0.251
Cephalic diameter ¬	30.00 (3)	30.50 (4)	27.105	0.109
Neurological Disability				
Yes	22 (1.5%)	2 (3.6%)	1467 º	0.226
No	1435 (98.5%)	54 (96.4%)		
Visual Impairment				
Yes	29 (2%)	2 (3.6%)	0.671 º	0.413
No	1428 (98%)	54 (96.4%)		
Hearing Impairment				
Yes	10 (0.7%)	0 (0%)	0.387 º	0.534
No	1447 (%)	56 (100%)		
Detection Type				
Intrauterine (during pregnancy)	327 (29.8%)	18 (39.1%)	1831 ^#^	0.176
Postpartum	771 (70.2%)	28 (60.9%)		
Magnetic resonance imaging results				
Normal result	4 (10%)	1 (100%)		
Altered result, suggestive of infection	28 (70%)	0 (0%)	7380 ^#^	0.061
Altered result, with other changes	6 (15%)	0 (0%)		
Indeterminate result	2 (5%)	0 (0%)		
Ultrasonography				
Normal result	116 (53.5%)	7 (58.3%)		
Altered result, suggestive of congenital infection	54 (24.9%)	3 (25%)	0.200 ^#^	0.978
Altered result, with other changes	21 (9.7%)	1 (8.3%)		
Indeterminate result	26 (12%)	1 (8.3%)		
Ultrasonography Transfontanela Result				
Normal result	209 (68.3%)	10 (71.4%)		
Altered result, suggestive of congenital infection	72 (23.5%)	3 (21.4%)	0.338 ^#^	0.953
Altered result, with other changes	19 (6.2%)	1 (7.1%)		
Indeterminate result	6 (2%)	0 (0%)		
CT scan result				
Normal result	237 (43.7%)	11 (44%)		
Altered result, suggestive of congenital infection	252 (46.5%)	11 (44%)	0.697 ^#^	0.874
Altered result, with other changes	46 (8.5%)	3 (12%)		
Indeterminate result	7 (1.3%)	0 (0%)		

(¬) Mann–Whitney U-test; (^#^) Pearson’s chi-square; (º) Fisher’s Exact Test; The missing values in the total sum of categories and groups correspond to the values omitted from the databases.

## Data Availability

Publicly available datasets were analyzed in this study. This data can be found here: https://datasus.saude.gov.br/nascidos-vivos-desde-1994/ (accessed on 15 November 2022).
